# Material Parameters Identification of Historic Lighthouse Based on Operational Modal Analysis

**DOI:** 10.3390/ma13173814

**Published:** 2020-08-28

**Authors:** Agnieszka Tomaszewska, Milena Drozdowska, Marek Szafrański

**Affiliations:** 1 Department of Structural Mechanics, Faculty of Civil and Environmental Engineering, Gdańsk University of Technology, Narutowicza 11/12, 80-233 Gdańsk, Poland; 2 Department of Rail Transportation and Bridges, Faculty of Civil and Environmental Engineering, Gdańsk University of Technology, Narutowicza 11/12, 80-233 Gdańsk, Poland

**Keywords:** material parameters identification, vibration measurements, testing, operational modal analysis, lighthouse, historic tower

## Abstract

In the present paper, the identification of the material parameters of a masonry lighthouse is discussed. A fully non-invasive method was selected, in which the material properties were determined via numerical model validation applied to the first pair of natural frequencies and their related mode shapes, determined experimentally. The exact structural model was built by means of the finite element method. To obtain experimental data for the inverse analysis, operational modal analysis was applied to the structure. Three methods were considered: peak picking (PP), eigensystem realization algorithm (ERA) and natural excitation technique with ERA (NExT-ERA). The acceleration’s responses to environmental excitations, enhanced in some periods of time by sheet piling hammering or by sudden interruptions like wind stroke, were assumed within the analysis input. Different combinations of the input were considered in the PP and NExT-ERA analysis to find the most reasonable modal forms. A number of time periods of a free-decay character were considered in the ERA technique to finally calculate the averaged modal forms. Finally, the elastic modulus, Poisson’s ratio and material density of brick, sandstone and granite masonry were determined. The obtained values supplement the state of the art database concerning historic building materials. In addition, the numerical model obtained in the analysis may be used in further cases of structural analysis.

## 1. Introduction 

The parameter identification of the materials used in historic structures demands the application of non-destructive means. The need to preserve structural safety and integrity is usually superior to any goal of structural testing. However, recognizing the properties of materials used in historic structures helps to select modern materials for some restoration works. The properties may be identified in the inverse analysis, in which the structural parameters are determined based on the measured structural response to a known, or unknown, action. The present paper discusses the identification of the material parameters of a slender masonry lighthouse. A fully non-invasive method was selected, in which the material properties were determined by the numerical model validation applied to the first pair of natural frequencies and the related mode shapes that were experimentally determined.

To obtain experimental data for the inverse analysis, operational modal analysis (OMA) was applied to the structure. OMA is widely discussed in the literature concerning structural health monitoring (SHM). Numerous OMA methods have been developed [1,2], and they are dedicated to certain vibration cases, i.e., free-decay or ambient. Their efficiency depends on the quality of the vibration signals. Subsequently, the quality of the OMA results determine the possibility of its further usage. Natural frequencies or modal forms, when identified with a considerable error, will induce untrue results or conclusions. Therefore, the quality of the OMA results must be treated with care. 

In the present paper, the modal identification (MID) of the lighthouse is discussed considering three kinds of structural excitation. The following MID techniques are applied herein: the eigensystem realization algorithm (ERA), the peak picking technique based on correlation analysis (PP-CA), and the natural excitation technique with ERA (NExT-ERA). The application of these three techniques increases the chance of true results being obtained. 

The considered tower is a historic lighthouse situated in Gdańsk (Poland). The brick construction is massive and rigid due to the spiral granite staircase filling the whole tower interior. The modal identification of similar structures is described in the literature [3]. Examples for masonry bell towers are presented in [4,5]. The SHM systems built for such structures are described in [6,7]. The systems monitor natural frequencies, and correlate them with temperature changes or with a day’s anthropogenic activity in the city. Ref. [8] concerns the identification of the foundation stiffness of the masonry tower, based on experimentally identified modal data.

The aim of this paper is to determine reasonable MID results for the lighthouse, in order to use these in the material parameter identification of the structural FEM model. The outcomes of three OMA methods under various excitations were compared, whereby the excitation caused by sheet piling hammering was a unique one. The opportunity to measure the lighthouse’s vibrations under such conditions arose during the modernization of the port quay in Gdańsk. The basic excitation was the environmental impact most affected by wind and river influences. The study presents the results of the PP-CA technique in the three following excitation cases: ambient, ambient enhanced by the ground vibrations enforced by the hammer action, and the two combined, i.e., ambient enhanced by the hammer action in selected time periods. The NExT-ERA results relate to the first and the third above-mentioned excitation kinds. The ERA technique was applied to the short-term free-decay parts of the ambient signals, as well as to the steady-state vibrations generated by the sheet piling hammering. In the first case, natural frequencies and related mode shapes and damping ratios were identified. In the second case, forced vibration frequencies and related response shapes were obtained. 

The provided results prove that in the case of a broad spectrum of excitations acting on a real structure, the OMA results obtained using different techniques may not be repeatable. To approach the true results, the analysis should be performed with care and skepticism. Furthermore, the application of a few OMA methods should be considered to ensure more objective inferences. Such an approach accepted in the described study provided reasonable MID results, which were further applied in the numerical model of validation for the lighthouse so as to finally determine the mechanical properties of the materials used in the construction, as well as their elastic support.

## 2. Materials and Methods 

### 2.1. The Lighthouse

#### 2.1.1. Description of the Structure

**Figure 1:**
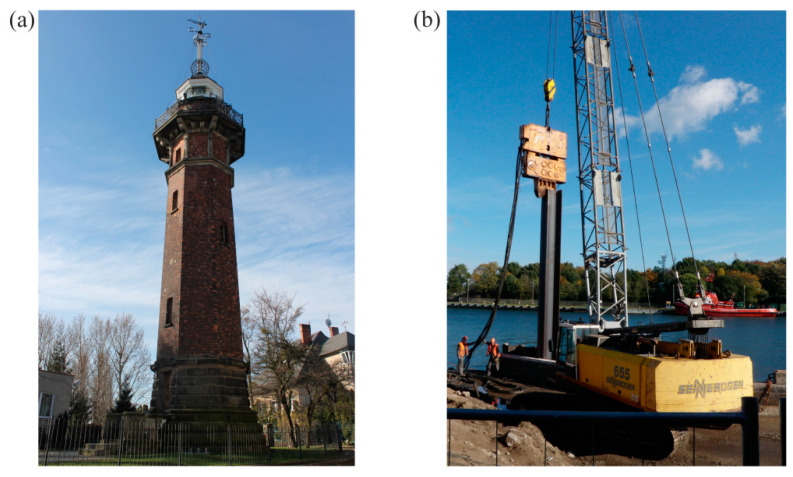
(**a**) the lighthouse; (**b**) sheet piling hammering next to the lighthouse.

The construction of the lighthouse (Figure 1a) was finished in October 1894. It is located approximately 50 m off the Dead Vistula River channel in the Port of Gdańsk across Westerplatte Peninsula.

The cross-section of the lighthouse is octagonal. It is 27.3 m high, with a masonry-build, load-bearing corpus of 22.5 m and a light room placed above it. There are two granite terraces at a height of 20.2 m and 22.5 m. The external diameter of the masonry part varies with height, from 6.8 m with a wall thickness of 1.04 m at the bottom to 4.2 m with a wall thickness of 0.68 m at the corpus top. A brick column in the tower’s center supports the spiral granite stairs. The external stairs, leading to the entrance, are also made of granite. The walls are mainly made of brick. Only the lower wall is made of sandstone, up to the height of 4.2 m. The light room is of a lighter construction than the tower corpus as it is built of steel beams, posts and sheeting with wood paneling.

During World War II, an external wall of the upper part of the construction and the deck were damaged. The tower was mostly repaired between the 1940s and 1950s. The most recent renovation took place in 2003. The building’s inventory was made including the foundation. The tower stands on a concrete slab and oaken piles. The ground conditions under the slab were not specified. The technical condition of the structure was recognized as good. There are no visible cracks in the structure, suggesting that the tower is somehow damaged.

#### 2.1.2. Dynamic Measurements

**Figure 2:**
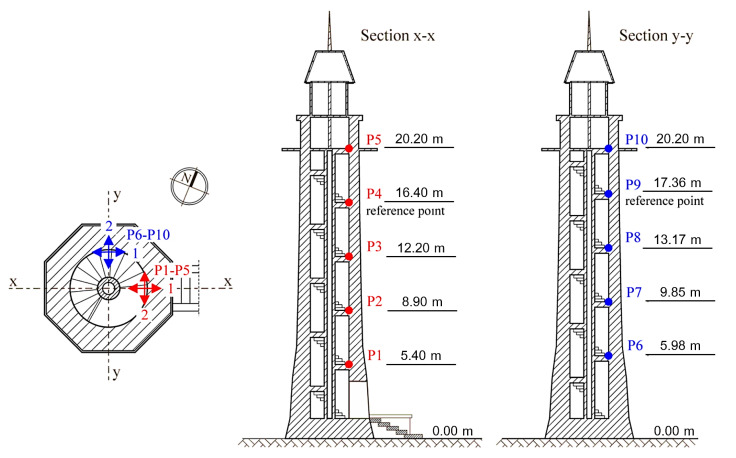
Measuring points’ locations (P1–P10) and directions *x* and *y* of measurements (sensors 1 and 2).

**Figure 3:**
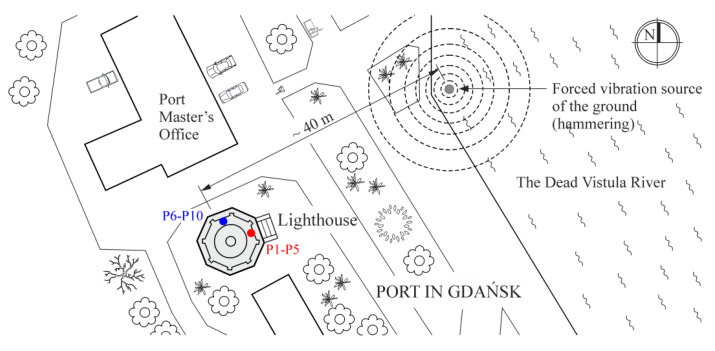
Situation plan.

The measurements were performed using a 16-bit HBM QuantumX acquisition system (Hottinger Baldwin Messtechnik GmbH, Darmstadt, Germany) together with one-dimensional piezoelectric accelerometers Isotron Endevco 7752-1000 (PCB Piezotronics Inc., Depew, NY, USA) with a voltage sensitivity of 1 V/g (±20%) and an amplitude response of 0.02–500 Hz (±5%). The sensors were located on the stairs along two vertical axes (points P1–P5 and points P6–P10 in the Figure 2 and Figure 3). The sensors locations were chosen based on the lighthouse’s geometry. The construction is nearly axisymmetric—the axial symmetry is disrupted by the windows and the entrance. In such structures, bending mode shapes appear as a pair of orthogonal vectors with very close natural frequencies. The vectors are aligned with ‘principal axes’ related to the details that disrupt the axial symmetry. It is reasonable to assume, then, that the first pair of (bending) modes is present in the directions weakened by openings in the walls.

Accelerations in two horizontal directions were measured at each point, i.e., perpendicular and parallel to the river’s axis (designated as directions *x* and *y* in Figure 2, respectively). Due to the equipment limitations, four series of measurements were carried out with the reference points P4 and P9.

**Figure 4:**
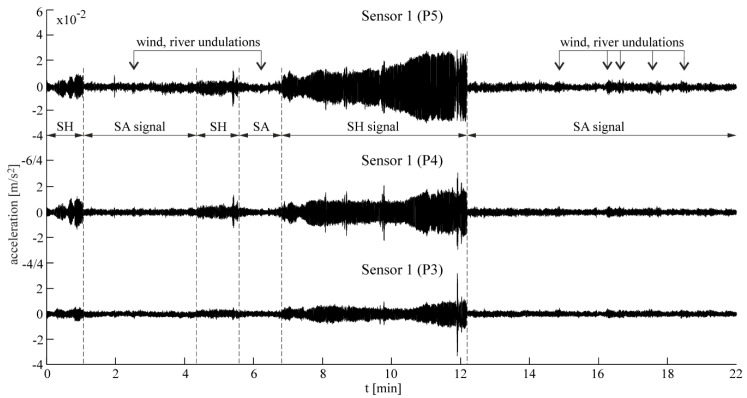
Selected acceleration time series with different types of signals—sensor 1 (P3–P5). Full length record is the SC signal.

The three following types of data were collected for further processing: SA—ambient vibration signals, SH—ambient vibrations amplified by vibratory hammer, and SC—combination of SA and SH signals. An explanation is given in Figure 4. The equal lengths of the signals in each group were considered as follows: SA and SC—1200 s, and SH—900 s, with a sampling frequency of 200 Hz. The SH signals were the shortest because of the limited time of hammering in subsequent measurement series. The location of the vibratory hammer in relation to the tower is shown in Figure 3. It operated at a setting of about 34 Hz.

### 2.2. Modal Identification Techniques 

#### 2.2.1. Peak Picking Method Based on the Correlation Analysis (PP-CA)

Ambient vibration responses to the (assumed) white noise signal are considered in the PP-CA method. The method is based on the correlation analysis of response signals in the frequency domain. This was proposed in [9] as the first OMA technique. Auto- and cross-correlation functions, determined for different measuring points together with the coherence functions, allow us to identify natural frequencies, as briefly described in [10]. Additional study of the phase shifts allows us to determine mode shapes. The damping ratios cannot be identified, which is the method’s limitation. The practical applications of the PP-CA technique are addressed in, e.g., [11,12,13].

#### 2.2.2. Eigensystem Realization Algorithm (ERA)

The eigensystem realization algorithm belongs to the group of direct, multi-input/multi-output system identification methods in the time domain [14]. This method evolved from the Ho-Kalmann minimum realization problem, and estimates the modal parameters, i.e., frequencies, damping ratios and mode shapes, based on free-decay, finite-time and noisy experimental data [15,16]. The method has been extensively discussed and used in various mechanical and engineering problems, e.g., machine construction optimization (see, e.g., [17]), vehicle-bridge dynamic interaction (see, e.g., [18,19]), and the damage detection and technical condition assessment of structures (see, e.g., [20,21]). The input is a free-decay structural response to a certain action or initial condition. In the present study, the environmental impacts are considered as the excitation source. Directly measured, short-time free-decay signals caused by blasts of wind or river undulations are considered as the input. A similar approach was applied in the paper [11], in which the ERA efficacy in the case of a massive masonry tower was studied.

#### 2.2.3. Natural Excitation Technique and ERA (NExT-ERA)

Free-decay data can also be obtained by applying a correlation analysis to the measured ambient vibration signals (assumed as stochastic). The correlation analysis transforms stochastic data into the deterministic domain, that is, into the correlation functions of decay character, which can be used as an input for the ERA algorithm. The natural excitation technique is this transformation [22]. The NExT-ERA methodology and applications were widely studied in the literature, e.g., [23,24,25,26], and are also used in the presented study.

### 2.3. Numerical Model of the Lighthouse

**Figure 5:**
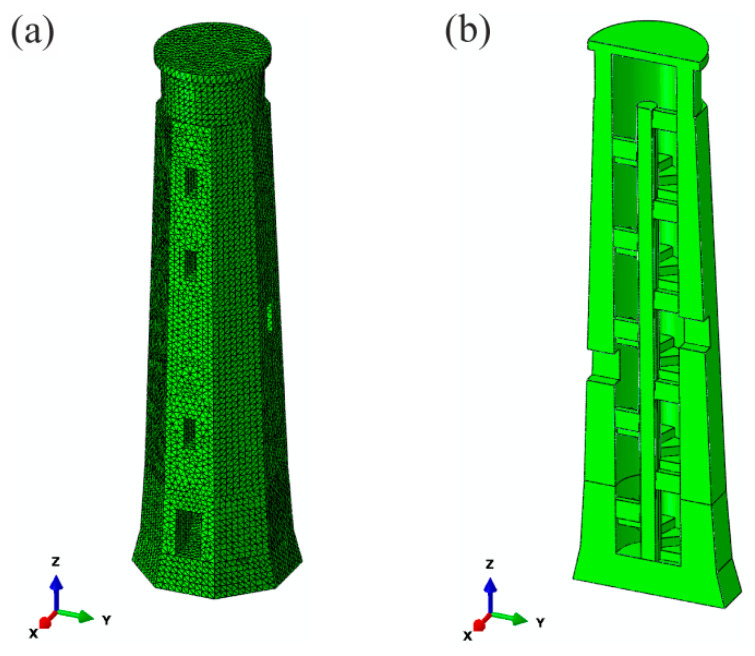
The finite element model of the lighthouse (Abaqus): (**a**) side view, (**b**) vertical section.

Based on the technical documentation and the tower inventory, a detailed numerical model of the lighthouse was created using the SIMULIA Abaqus FEA 2019 software [27] and the finite elements method (FEM) (Figure 5). The model contains brick walls, an inner granite staircase, brick columns and a sandstone base. The light room part was intentionally omitted, with it having a negligible influence on the modal parameters of the structure’s corpus (its mass accounts for 0.42% of the entire structural mass). A 10-node volume element (quadratic tetrahedron) with three translational degrees of freedom at each node (element C3D10) was chosen to generate a mesh. Overall, the model consists of 223,458 nodes and 146,358 elements in its entirety.

The homogeneous linear elastic material model is accepted for all materials. Mass density values were assumed on the grounds of the Polish design standards PN-EN ISO 12524:2003, PN-EN ISO 69446:1999 and PN-91/B-02020. The initial elastic modulus of brick masonry was chosen according to PN-EN 1996-1-1. The elastic moduli of sandstone and granite were selected based on Ref. [28]. It was suspected that the granite stairs were rigid and played the major role in lighthouse dynamic behavior, so the initial value was high. The sandstone base was also predicted to be stiff, however there were no cracks in the construction, suggesting little difference between the brick and sandstone parts, thus the value chosen was of a lower range. Initially, the model was fixed at the tower’s footing, but the support conditions were modified during the model’s calibration, and vertical linear spring elements were included to model the elastic support of the lighthouse. Horizontal support conditions were assumed to be rigid. 

## 3. Results

### 3.1. Modal Identification 

**Figure 6:**
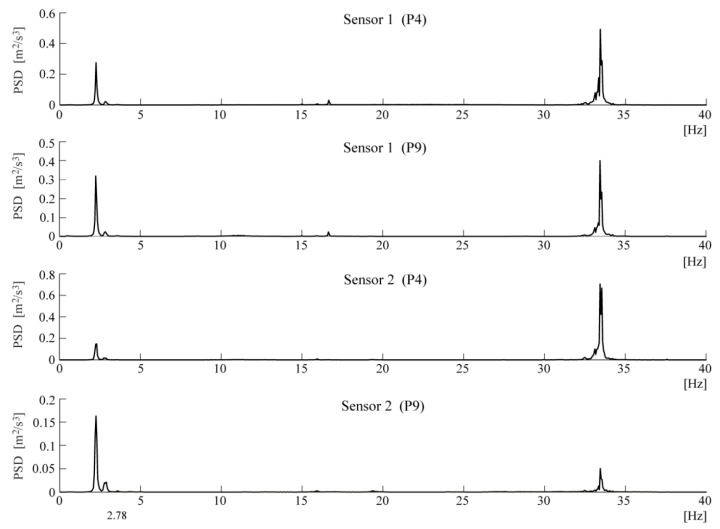
Selected spectra of acceleration records of the combined signals (SC).

**Figure 7:**
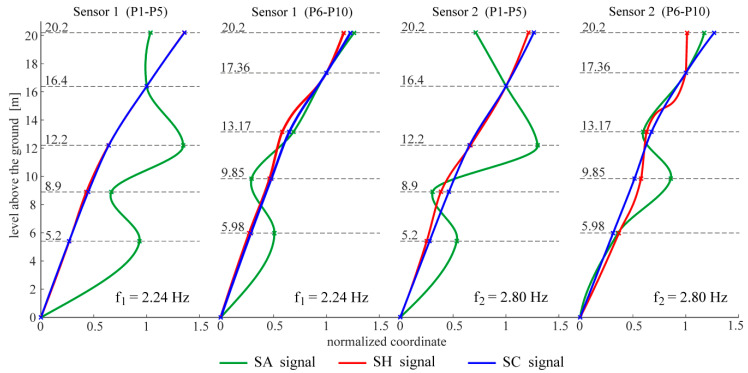
Mode shapes obtained via the PP-CA method for the three kinds of input signals.

**Figure 8:**
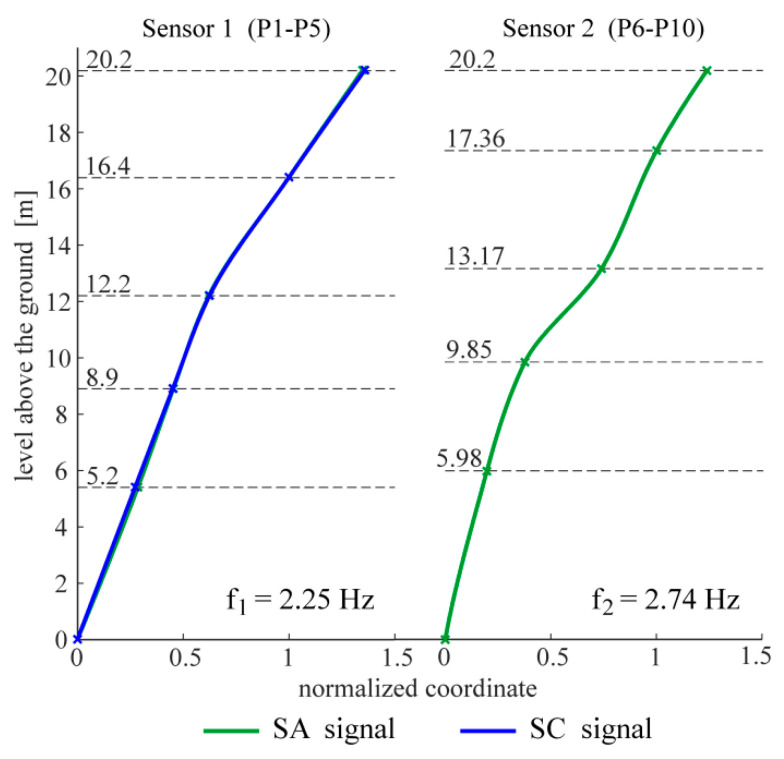
Mode shapes obtained via the NExT-ERA algorithm for the two kinds of input signals.

**Figure 9:**
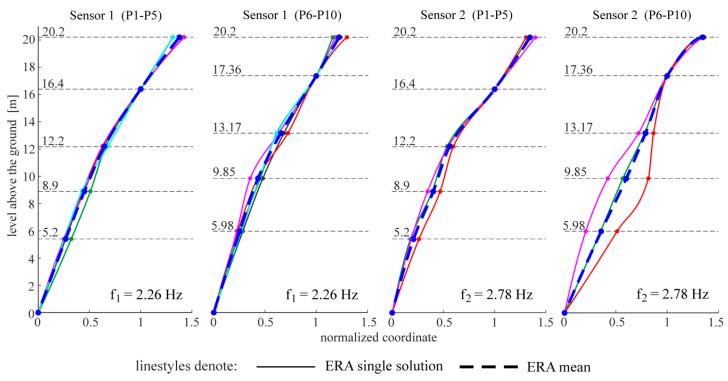
Mode shapes obtained via the ERA for different free-decay parts of the SA signals and the average result.

**Figure 10:**
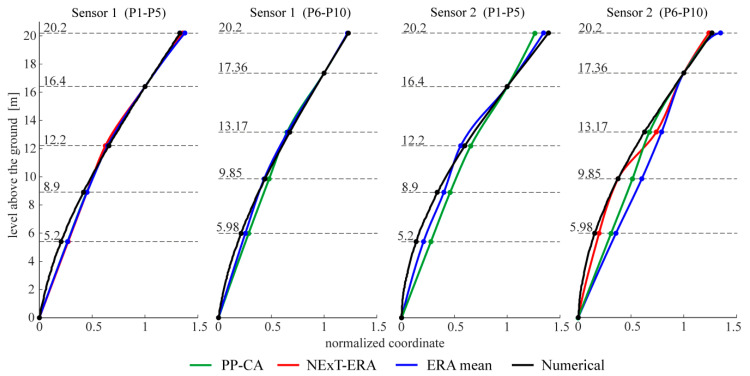
The comparison of the mode shapes obtained using different methods together with numerical results. PP-CA—the results based on SC signals, NExT-ERA—the results based on SA signals.

**Figure 11:**
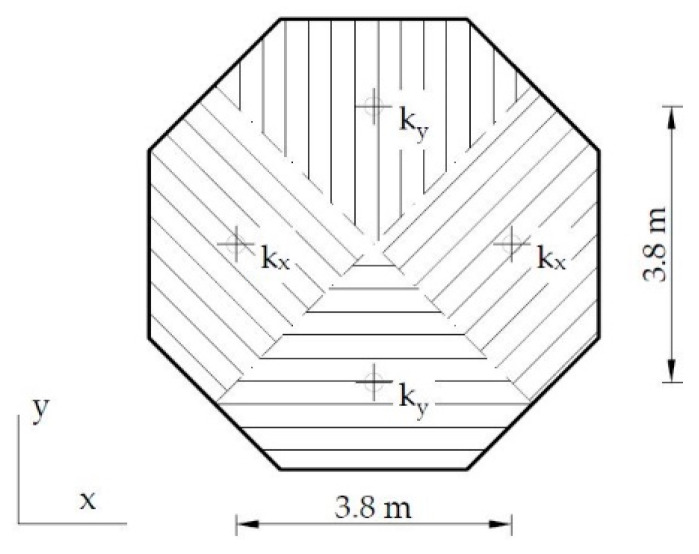
Supporting springs layout.

The following figures present the obtained results. Selected response spectra are presented in Figure 6. Subsequently, the mode shapes obtained from the PP-CA method for the three kinds of the input signals are plotted in Figure 7. Similar results of the NExT-ERA realization for the SA and SC signals are presented in Figure 8. The accepted modal amplitude coherence (MAC) criterion (95%) allowed us to determine only two mode shape vectors. Selected ERA solutions for different time intervals of ambient responses (SA signals) excited by blasts of wind or river undulations (see Figure 5) are presented in Figure 9, together with the average solution. The MAC criterion at a level of 98% is accepted. In the case of direction 1 (perpendicular to the river), five different parts of visible amplitudes and decay character were selected, while in the case of direction 2 (parallel to the river) three parts of signals could be selected. Signals between 4 s and 8 s in length, depending on the visible amplitude level, were processed by ERA. The considered lighthouse has a cantilever-type structure, so the prediction of the modal forms is intuitive. Thus, the most reasonable results of PP-CA and NExT-ERA were selected and put together with the theoretical forms and the average ERA results. Juxtaposition is presented in Figure 10. The summary of the identified natural frequencies and damping coefficients is presented in Table 1. All the presented results refer to the first pair of natural frequencies, visible in the spectra in Figure 6 (2.26 Hz, 2.78 Hz). However, a reasonable peak is also observed for the frequency 32.46 Hz, which corresponds to the forced vibrations caused by the vibratory hammer action. The related response shape was identified by the ERA and the NExT-ERA techniques. The results are presented in Figure 11, with a similar numerical mode shape.

### 3.2. FEM Model Validation

The primary FEM model, including a fixed support and the initial material parameters, produced the first pair of natural frequencies with the values of 6.96 Hz and 7.01 Hz, in the *x* and *y* directions, respectively. Such values were approximately three-fold higher than the measured ones. Thus, the boundary conditions were modified by adding vertical springs, and the model was manually calibrated to the natural frequencies and mode shapes obtained with the PP-CA technique in the combined signal variant. The following parameters of the model were validated: material parameters (elastic modulus and mass density) and the supporting spring stiffness. Four supporting springs were considered. The bottom surface of the base was divided into four areas, in which nodes were kinematically bounded in the *z* direction to the spring situated in this area (see Figure 11). 

The initial values were updated to obtain a sufficiently high compliance between numerical and experimental frequencies and mode shapes. The initial and final material parameters are summarized in Table 2. The initial stiffness of the supporting springs was *k_x_* = 1 × 10^10^ N/m and *k_y_* = 2 × 10^10^ N/m. The final stiffness of the springs was *k_x_* = 3.75 × 10^9^ N/m and *k_y_* = 3.75 × 10^10^ N/m. Table 1 compares the numerical and experimental frequencies of the lighthouse. 

## 4. Discussion

The identification of the material parameters of a real historic structure, based on modal identification results, is discussed in this paper. The efficacy of the modal identification is crucial in obtaining true material parameters. The efficacy appeared to be different for the natural frequencies and the mode shapes. The three different methods considered in this study provide convergent results for the natural frequencies. The values are between 2.24 and 2.26 Hz, and 2.74 and 2.80 Hz, for the first pair of natural frequencies (see Table 1). Both modes are the first two bending mode shapes in two orthogonal directions. The obtained values of the fundamental frequencies are similar to the characteristics of other masonry towers. In the paper [29], the values for 30 masonry towers with heights of 16–46 m are summarized. They are between 0.61 Hz and 5.28 Hz. The damping coefficient corresponding to the first natural frequency varies between 0.0226 and 0.0244. The values are included in the most common range of damping coefficient determined for masonry towers, which is 0.02–0.03, according to [30]. The mode shapes identified by the application of the three OMA techniques to the three kinds of input signals are not always repeatable. First of all, the PP-CA method, which is dedicated to ambient excitations, does not produce a reliable mode shape for the SA signals (see Figure 7). The application of the ambient signals enhanced by the sheet peel hammering significantly improves the result, however untypical changes in the mode shapes curvatures are visible in the upper parts of the modes. The most reasonable results were obtained for the combined signals, examples of which are presented in Figure 4. In this case, the mode shapes show the curvature of one sign along the whole length; moreover, they show the typical shapes of the modes of a cantilever supported by a rotational spring—such a simplified model can approximate the considered tower. It is worth mentioning that the efficacy of the mode shapes’ identification does not relate to the signal amplitudes in general. The average root mean square (RMS) of all signals in subsequent groups equals 0.0014 for SA, 0.0039 for SH and 0.0027 for SC signals. The SH signals have the biggest amplitudes, related to the hammering frequency. The identification of modes related to other frequencies is enhanced somehow, but the best solutions are obtained when the combined signals are considered with moderate RMS and with different dynamic influences. Limited results are obtained by the NExT-ERA technique. Only two modal vectors are obtained based on the SA signals, and both of them are in directions perpendicular to the wall planes (Figure 8). The SH signals produced no results, and the SC signals provide only one modal vector in the *x* axis of the tower (Sensors 1, P1–P5 line). Contrary to the PP-CA case, the application of SC signals does not improve the results obtained for the SA signals with the NExT-ERA algorithm. The different efficiencies of various OMA techniques in the practical cases of six British lighthouses are also reported in the paper [3].

The application of the ERA technique to the short-term free-decay parts of SA signals leads to a set of modal forms. Then, the averaged mode shape vector is found as the final ERA solution. The most considerable results obtained using three identification methods are compared in Figure 10, together with the numerical results. The PP-CA results were obtained for the case of the SC signals, while the NExT-ERA results were obtained for the SA signals. The comparison of the results shows the most repeatable forms in the line P1–P5, measured by sensor 1. This suggests the best quality of the signals collected by these sensors. The line is situated along the tower wall on the river side, and sensor 1 measures the accelerations in the direction perpendicular to the river (direction *x*, see Figure 2). This line and these sensors receive the highest excitation from the river’s undulations. This may be the factor affecting the signal’s quality. Besides, the structure looks integral, and no damages are visible. However, maybe the renovations after World War II play some role in the signal deteriorations. It is worth mentioning that the different lengths of signal considerations in the NExT-ERA and the ERA techniques did not improve the presented results. 

The enhanced OMA analysis validated the data for the FEM model and the material parameters’ identification via the validation process. The results obtained and presented in Table 2 are included in the range of material parameters reported for other masonry structures. In the papers [4,31,32], the investigations of masonry bell towers are described. The elastic modulus of the masonry tower described in [4] was assessed by a simplified global analysis based on the first natural frequencies obtained through experimental analysis. The results were, depending on direction, 3.579 and 4.746 GPa, with frequencies of 1.294 and 1.489 Hz. However, in the FE model the elastic modulus value of 2.819 GPa was implemented. A similar tower is the object of another study [31], where the elastic modulus is stated as 1.8 GPa with a first frequency of 0.67 Hz. In the paper [32], the elastic modulus of masonry varied from 1.8 GPa to 2.5 GPa, relative to the wall height. The first frequencies were assumed as 0.585 Hz and 0.709 Hz. In every case, stairs were unimportant from a structural point of view, and were thus not included in any numerical model. According to [28], the elastic modulus of granite varies from 2.59 GPa to 88.79 GPa, and in the case of sandstone from 3.4 GPa to 71.7 GPa. In another paper [33], 48.8 GPa is the identified value of the elastic modulus of stone masonry. The values of the elastic moduli of granite masonry applied in three different historic structures are presented in [34]. They belong in the range of 20.8–39.2 GPa, with a unit weight in the range of 24.1–26.4 kN/m^3^. All those values are similar to the material parameters identified in the present study.

**Figure 12:**
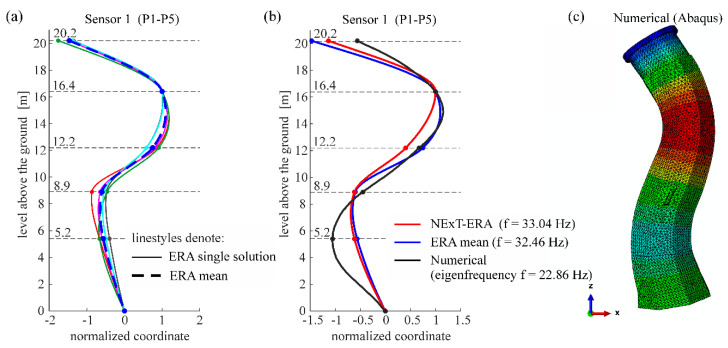
The response shape induced by the peel hammering and similar numerical mode shape: (**a**) ERA results; (**b**) experimental response shape (NExT-ERA and ERA mean results) in comparison with numerical third mode shape (**c**) the third numerical bending mode shape (Abaqus).

An interesting and rare result concerns the shape response identification, which is induced by the sheet peel hammering (see Figure 12). This shape could be identified for the *x* direction only (perpendicular to the river), which corresponds to the direction of wave propagation in the ground generated by the hammer action. This particular response could be identified using the ERA and the NExT-ERA methods. This shape is similar to the eigenmode of the numerical model (third bending one) (see Figure 12). The related numerical and experimental frequencies differ considerably. The distant values of the frequencies prove the safety of the peel hammering works in the vicinity of the lighthouse.

## 5. Conclusions

The presented study proves that in the case of a real structure with a limited possibility of dynamic excitation, reliable mode shape identification is a complex issue. Such a task demands the engagement of a few OMA techniques to authenticate the identified modal forms. The importance of the careful OMA inference is emphasized when the results are applied in the mathematical model of structural identification. The analysis provided the information concerning the mechanical parameters of the materials used in the considered lighthouse construction. The results complement the knowledge concerning such parameters for historic building materials.

## Figures and Tables

**Table 1:** Natural frequencies and damping coefficient identified by different methods.

Method	Natural Frequency—First Mode (Hz)	Natural Frequency—Second Mode (Hz)	Damping Coefficient
Peak Picking	2.24	2.80	-
NExT-ERA	2.25	2.74	0.0226
ERA	2.26	2.78	0.0244
Numerical	2.23	2.81	-

**Table 2:** Initial material parameters and parameters obtained by FEM model updating.

Material	Initial Elastic Modulus (GPa)	Final Elastic Modulus (GPa)	Poisson’s Ratio	Initial Density (kg/m^3^)	Final Density (kg/m^3^)
Brick masonry	1	2.4	0.167	2200	2100
Sandstone	10	2.4	0.2	2400	2100
Granite	80	26	0.3	2600	2000
